# Supplementary material for: Deep Transcriptomics Reveals Cell-Specific Isoforms of Pan-Neuronal Genes
Source: bioRxiv. 2024 May 20:2024.05.16.594572. Preprint. [Version 1] doi: 10.1101/2024.05.16.594572 (PMC11142100; doi:10.1101/2024.05.16.594572)
Supplement: Supplement 7 [file NIHPP2024.05.16.594572v1-supplement-7.pdf]

bioRxiv preprint doi: https://doi.org/10.1101/2024.05.16.594572; this version posted May 20, 2024. The copyright holder for this preprint (which was not certified by peer review) is the author/funder, who has granted bioRxiv a license to display the preprint in perpetuity. It is made available under aCC-BY 4.0 International license.

# SUPPLEMENTAL FIGURES

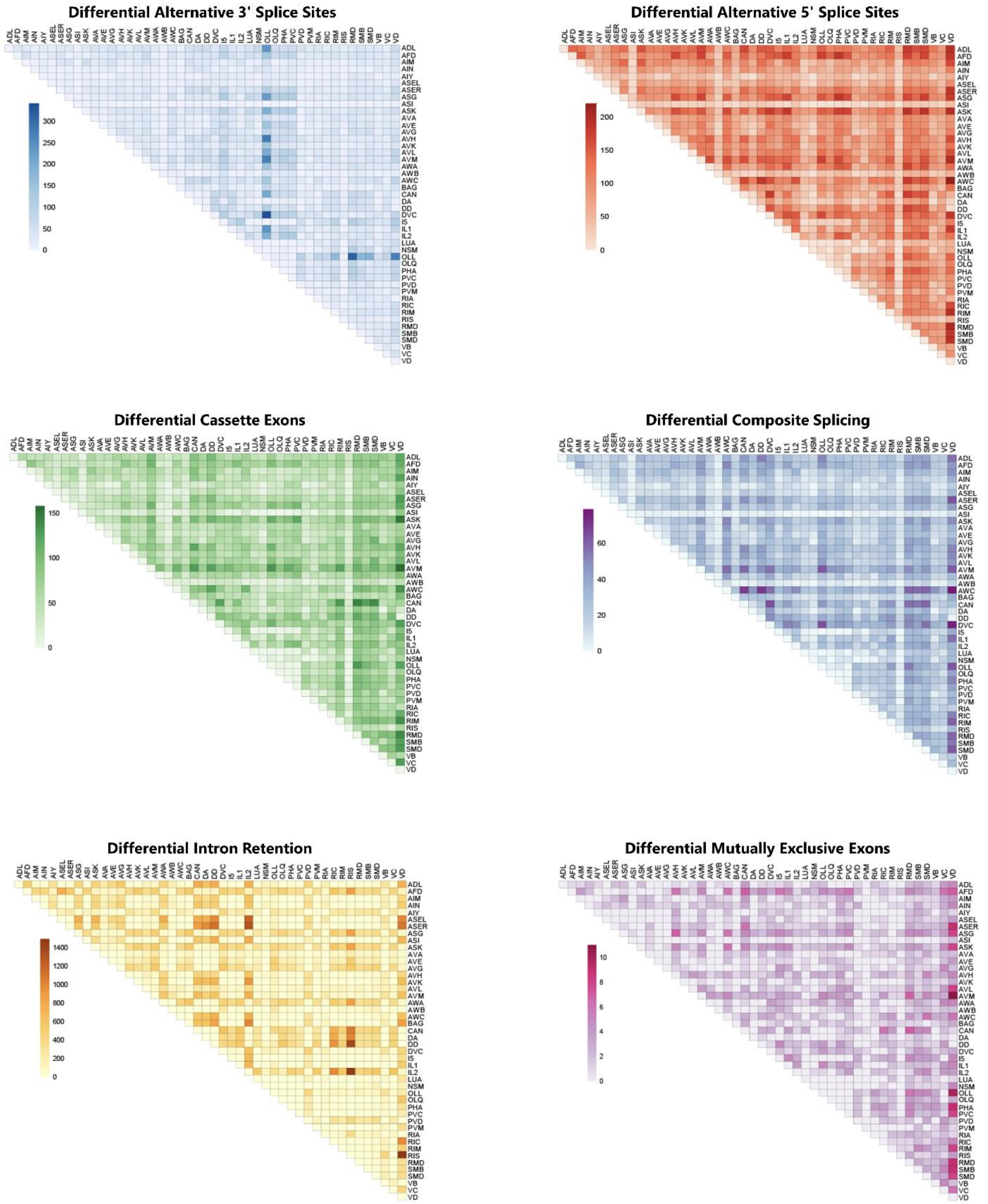


**Figure S1: Alternative splicing across neuronal cell types revealed by deep transcriptomes.** Heatmaps, as in Figure 1E, showing number of differential alternative splicing events across all pairwise comparisons for all classes of alternative splicing detectable by JUM (alternative 3′ splice site, 5′ splice site, cassette exons, intron retention, mutually exclusive exons, and composite splicing).

bioRxiv preprint doi: https://doi.org/10.1101/2024.05.16.594572; this version posted May 20, 2024. The copyright holder for this preprint (which was not certified by peer review) is the author/funder, who has granted bioRxiv a license to display the preprint in perpetuity. It is made available under aCC-BY 4.0 International license.

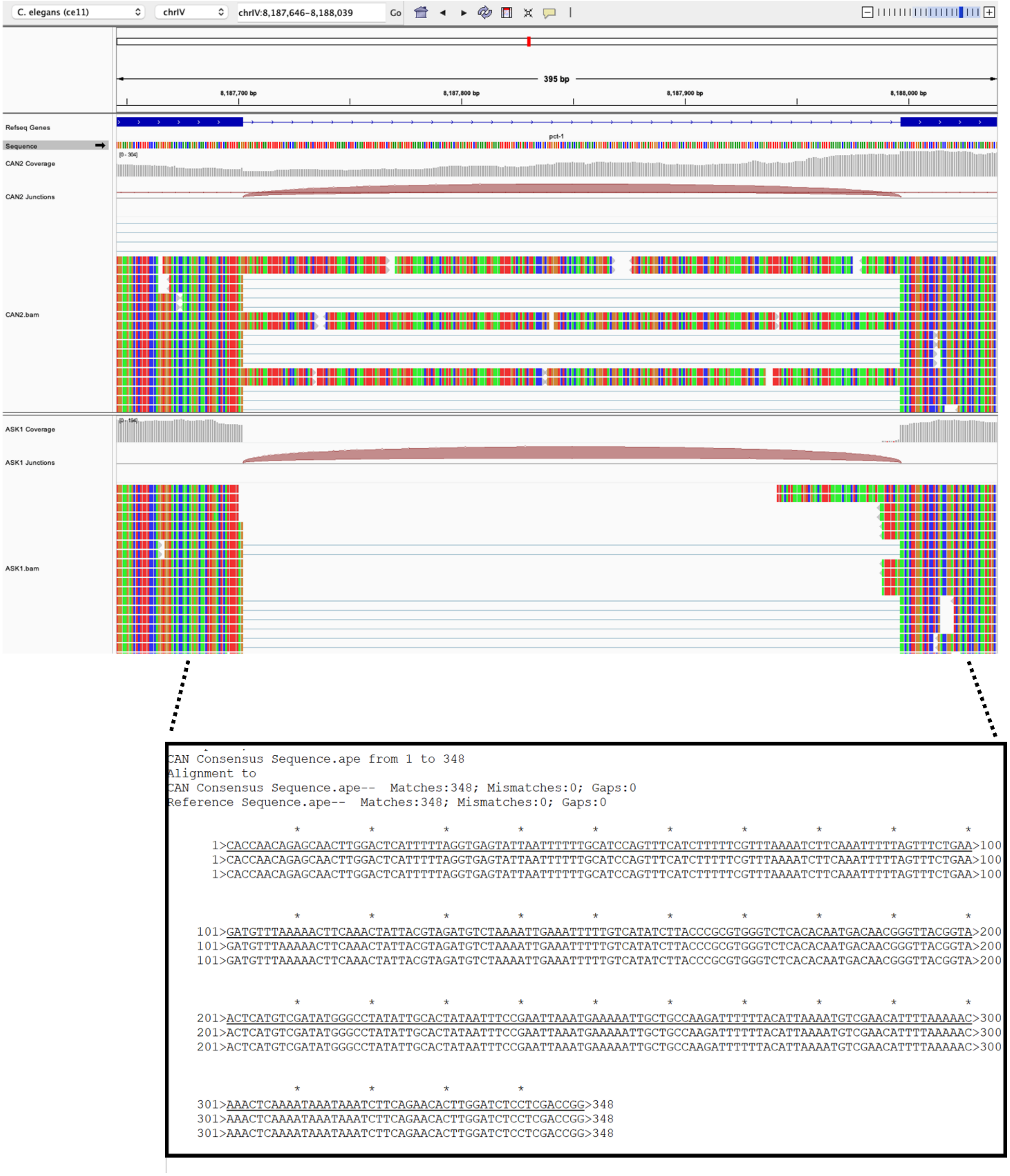


**Figure S2: Intron retention of *pct-1* in CAN neuron is not a consequence of strain-specific mutations.** Visualization of sequencing reads via IGV showing no mutations in either CAN or ASK neuron RNA Seq (lower tracks) compared to each other, or to reference sequence (upper track). Bottom panel is a sequence alignment between the annotated nucleotides and intron-retained sequenced nucleotides showing no mutations observed.

bioRxiv preprint doi: https://doi.org/10.1101/2024.05.16.594572; this version posted May 20, 2024. The copyright holder for this preprint (which was not certified by peer review) is the author/funder, who has granted bioRxiv a license to display the preprint in perpetuity. It is made available under aCC-BY 4.0 International license.

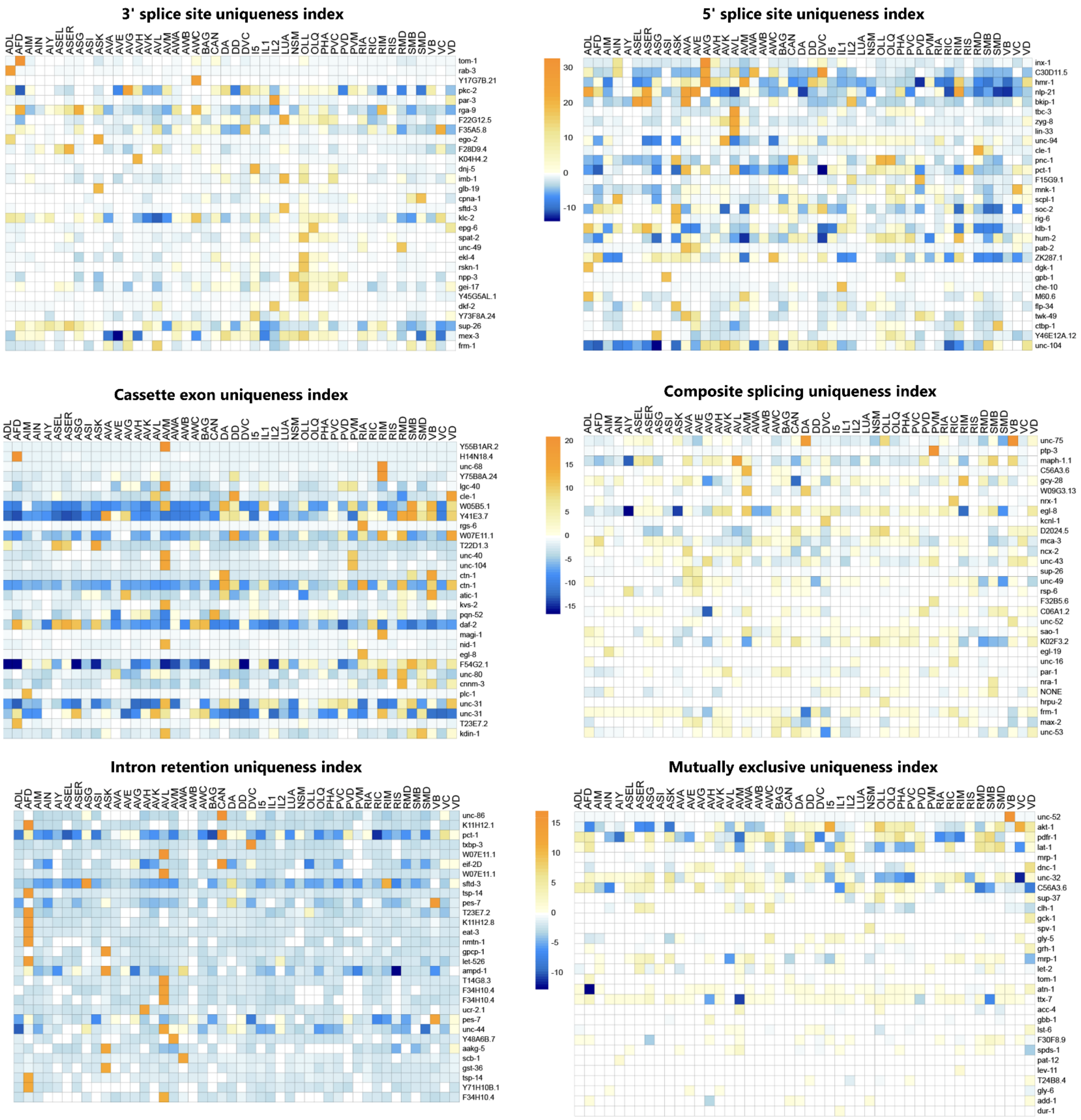

bioRxiv preprint doi: https://doi.org/10.1101/2024.05.16.594572; this version posted May 20, 2024. The copyright holder for this preprint (which was not certified by peer review) is the author/funder, who has granted bioRxiv a license to display the preprint in perpetuity. It is made available under aCC-BY 4.0 International license.

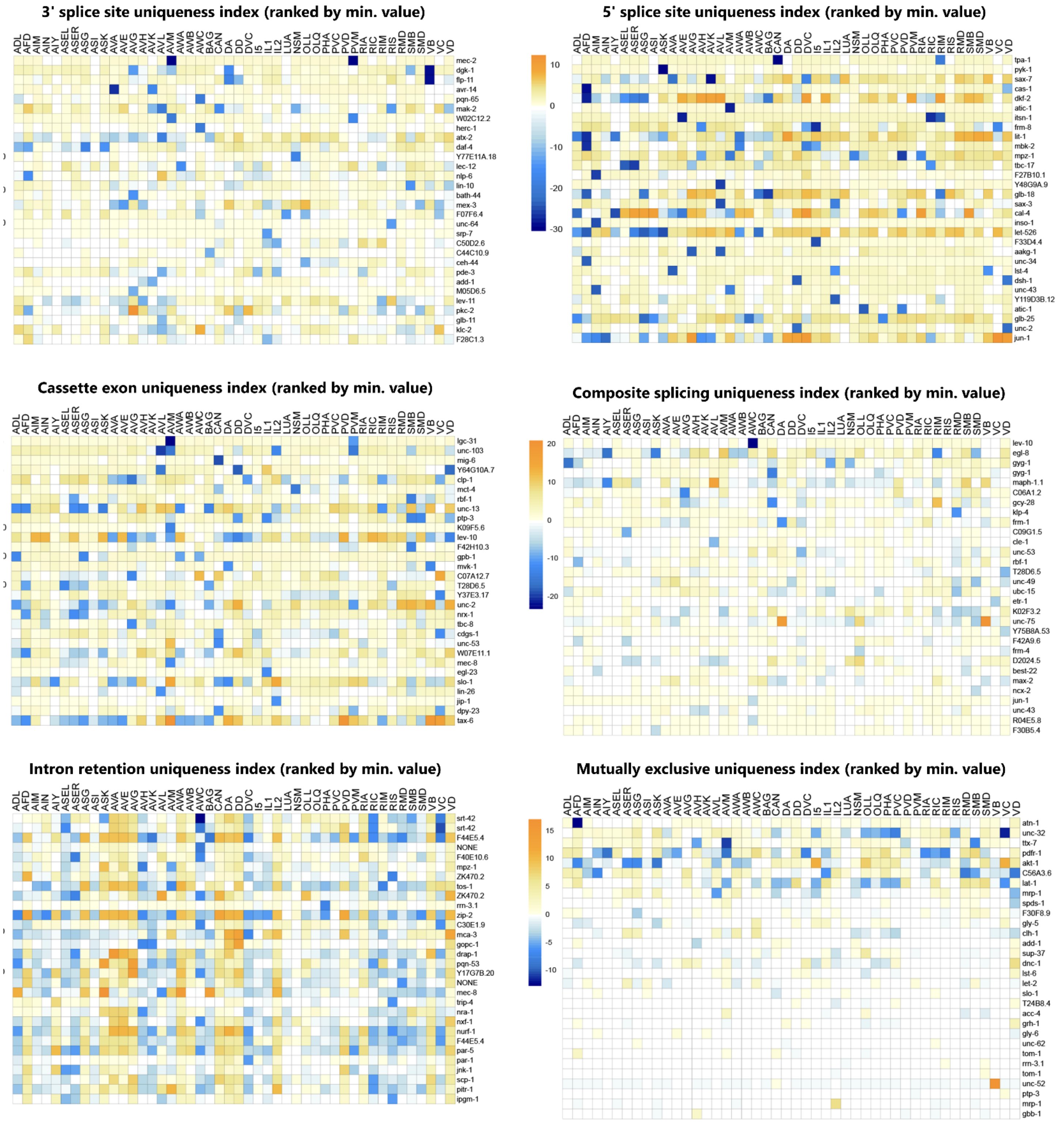


**Figure S3: Uniqueness index values for all alternative splicing types.** Top 30 uniqueness values, upper 6 panels are sorted for highest positive values (corresponding to high PSIs and/or upstream splice site selection), lower 6 panels for most negative values (corresponding to low PSIs and/or downstream splice site selection).

bioRxiv preprint doi: https://doi.org/10.1101/2024.05.16.594572; this version posted May 20, 2024. The copyright holder for this preprint (which was not certified by peer review) is the author/funder, who has granted bioRxiv a license to display the preprint in perpetuity. It is made available under aCC-BY 4.0 International license.

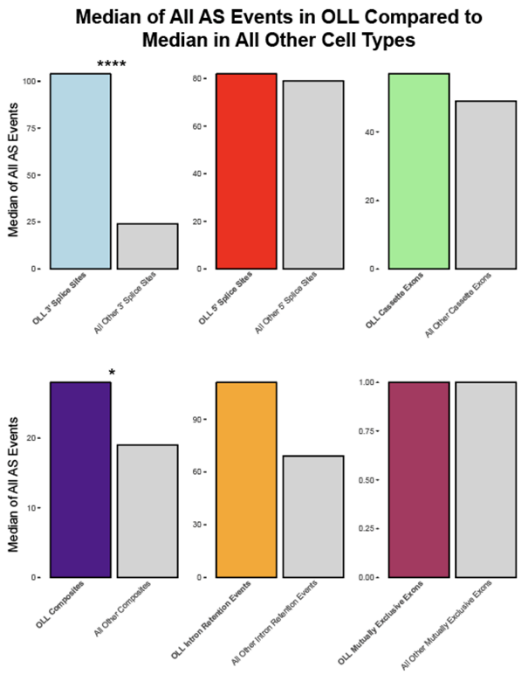


**Figure S4: OLL is uniquely enriched for differential 3′ splice site selection.** Median values of number of differential alternative splicing events between OLL and all other neurons (left, colored bars) versus all other neuron pairwise comparisons (right, gray bars). OLL has much more alternative 3′ splice site selection compared to other neurons, but this is not the case for other types of alternative splicing. *** = t-test, $p<0.001$.

bioRxiv preprint doi: https://doi.org/10.1101/2024.05.16.594572; this version posted May 20, 2024. The copyright holder for this preprint (which was not certified by peer review) is the author/funder, who has granted bioRxiv a license to display the preprint in perpetuity. It is made available under aCC-BY 4.0 International license.

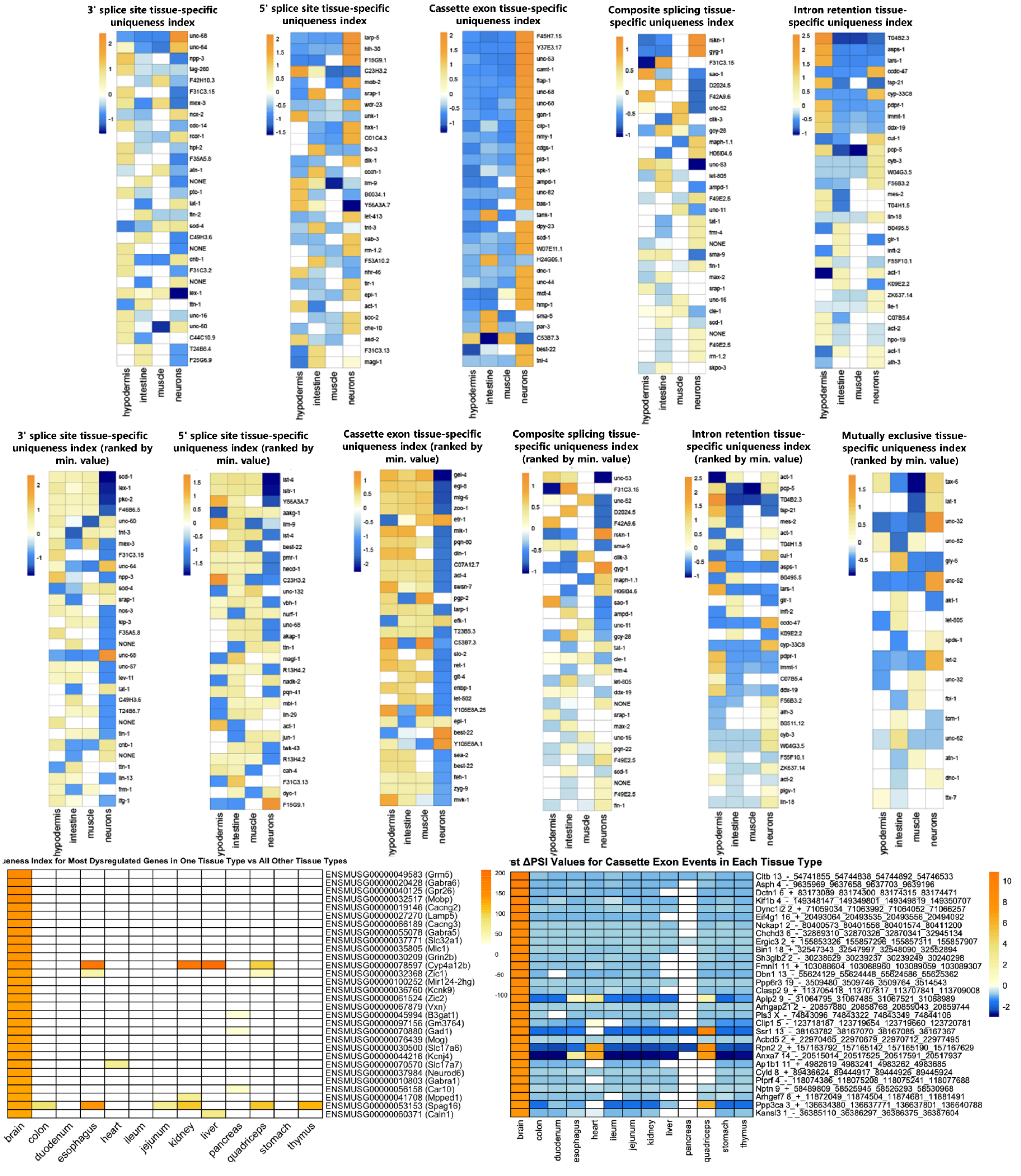


**Figure S5: Uniqueness index values for tissue-specific alternative splicing in worm and mouse.** Upper panels, Heatmaps as in Figure 5B but expanded to top 30 splicing events. Upper panels are sorted for highest positive values (corresponding to high PSIs and/or upstream splice site selection), lower panels for most negative values (corresponding to low PSIs and/or downstream splice site selection). Mutually exclusive exons are presented only once because, due to their small number, all of the top values (both positive and negative)

bioRxiv preprint doi: https://doi.org/10.1101/2024.05.16.594572; this version posted May 20, 2024. The copyright holder for this preprint (which was not certified by peer review) is the author/funder, who has granted bioRxiv a license to display the preprint in perpetuity. It is made available under aCC-BY 4.0 International license.

appear on a single heatmap. Lower panels, uniqueness index values for tissue-specific mouse data. Left side, brain-specific gene expression profiles. Right side, brain-specific cassette exons uniquely included in brain compared to all other tissues.

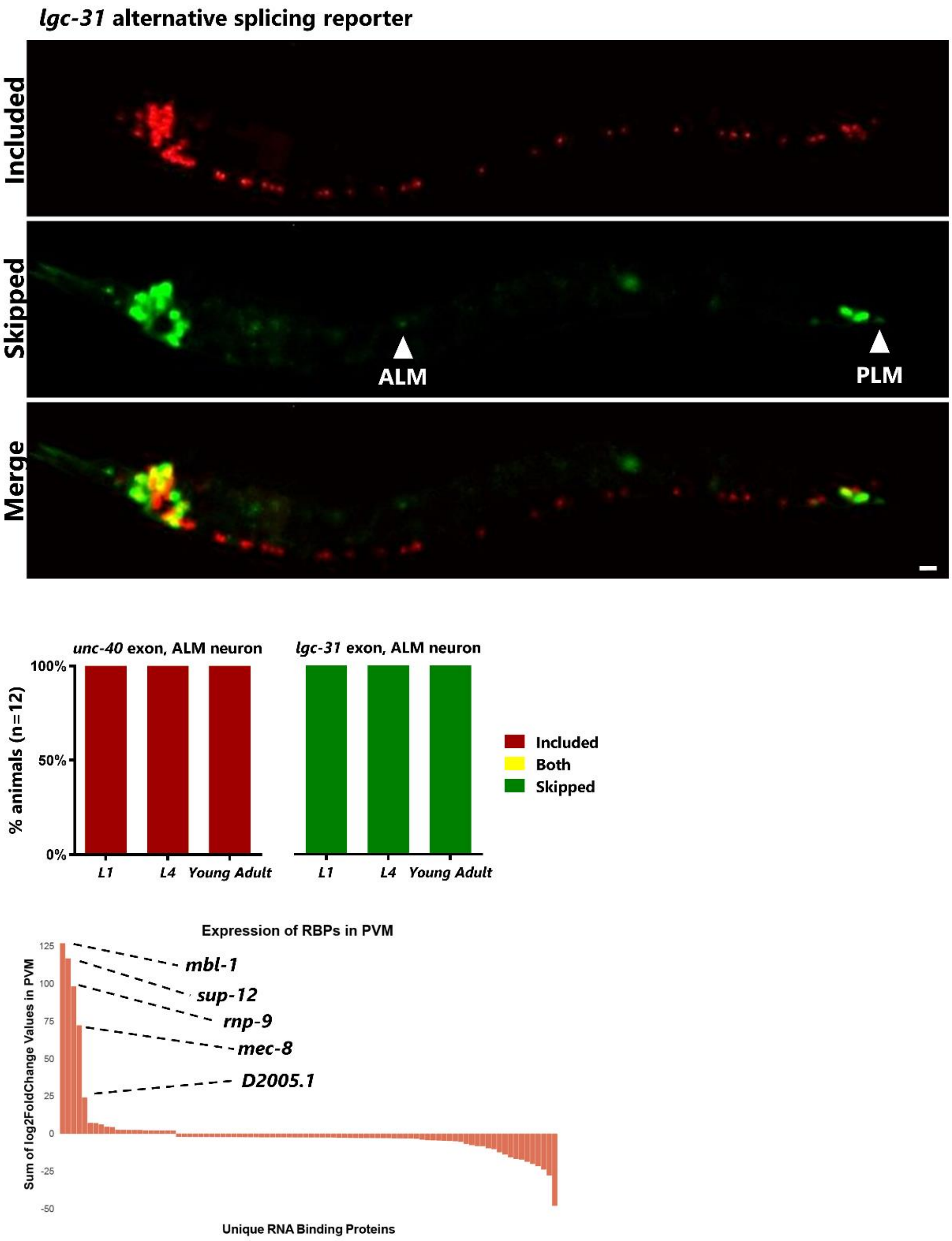


**Figures S6: Unique alternative splicing and RNA Binding Protein expression in touch neurons.** Upper panel, *lgc-31* splicing reporter, as in 6C for the *unc-40* splicing reporter, shows alternative splicing unique to touch neurons (exon skipping, GFP) compared to many other neurons (exon inclusion, RFP). Scale bar

bioRxiv preprint doi: https://doi.org/10.1101/2024.05.16.594572; this version posted May 20, 2024. The copyright holder for this preprint (which was not certified by peer review) is the author/funder, who has granted bioRxiv a license to display the preprint in perpetuity. It is made available under aCC-BY 4.0 International license.

represents 10 µm. Middle panel, splicing phenotypes for *unc-40* and *lgc-31* cassette exons are invariant across individuals and across developmental stages (ALM neuron selected due to ease of unambiguous identification). Lower panel, RNA binding protein uniqueness expression levels, as in Figure 6D for AVM, but shown here for PVM.
